# Embodied Effects in Verb Processing and Learning: A Three-Level Meta-Analysis

**DOI:** 10.3390/bs16060914

**Published:** 2026-06-03

**Authors:** Yueyan Huang, Yihui Zhai, Zhujun Jiang, Yihan Wang, Li Li

**Affiliations:** 1 School of Foreign Studies, South China Normal University, Guangzhou 510631, China; 2024010125@m.scnu.edu.cn (Y.H.); xzldyj@163.com (Y.Z.); 2 Key Laboratory of Chinese Learning and International Promotion, School of International Culture, South China Normal University, Guangzhou 510631, China; zhujunne@163.com; 3 School of Education, The Johns Hopkins University, Baltimore, MD 21218, USA; qryihanwang0202@163.com

**Keywords:** verb processing, verb learning, embodied effect, three-level meta-analysis, moderating effect

## Abstract

Verbs describe actions and changes in states, and are closely grounded in perceptual and motor experiences. Their core attributes align precisely with the embodied effect, which has increasingly attracted attention in research on verb processing and learning. To further clarify the influencing mechanisms and potential factors (verb concreteness, motor features, verb layer, depth of processing task and language status) of the embodied effect in verb processing and learning, a three-level meta-analysis with a random-effects model was performed on 37 studies (265 effect sizes and 12,357 participants) published in the past 15 years. The results revealed that there was an embodied effect in verb processing and learning, with a significant total effect size (Hedges’ g = 0.259, *p* < 0.005). Specifically, verb concreteness and verb layer moderated this effect. These findings suggest that future research should further explore the mechanisms and interactive influences of verb features and contextual information on the embodied effect. The findings further imply that language instruction may benefit from integrating embodied experiences with contextualized linguistic input to enhance verb learning and comprehension.

## 1. Introduction

How embodied cognition shapes verb processing and learning has been a hot topic in cognitive linguistics and neuroscience in recent years, with its potential factors also meriting special attention. According to the embodied cognition framework, our body is the foundation for how we perceive and experience the world, and it plays a key role in language processing. Thus, language comprehension and production are not merely abstract cerebral activities but rely on multimodal experiences that engage the whole body (Lu & Yang, 2025). Meanwhile, verbs are the core of a sentence connecting subjects and objects, whose semantics links closely to bodily experiences (Lakoff & Johnson, 1980; Zhong, 2022; Rolán et al., 2023). Therefore, verbs have become the focus of research on language’s embodied effects.

Numerous studies on verb processing and learning provide behavioral and neuroscientific evidence for embodied cognition. Glenberg and Kaschak (2002) reported the Action-Sentence Compatibility Effect (ACE), whereby participants respond faster to verbs when the implied action direction aligns with the required response, supporting the motor system’s role in language comprehension. Embodied cognition proposes that understanding action-related verbs engages the sensorimotor system. Neuroimaging studies using fMRI and ERP have shown that processing these verbs activates brain regions such as the primary motor cortex, premotor cortex, and other related areas (Pulvermüller & Fadiga, 2010; Raposo et al., 2009; Van Dam & Desai, 2017; X. Li et al., 2022). Despite robust evidence, consensus is lacking regarding the involvement of bodily experience in verb processing and learning (Dalla Volta et al., 2014; Van Dam & Desai, 2017). Some studies report no embodied effects: Postle et al. (2008) found that processing specific action words did not activate primary or premotor areas, and Innocenti et al. (2014) observed no motor simulation during abstract verb processing. More recently, Zappa et al. (2024) combined EEG with virtual reality to examine embodied learning of L2 action verbs. No motor resonance was observed in the mu and beta bands for the highly embodied learning group, suggesting that short-term training may be insufficient to form stable motor representations. These findings partially support traditional cognitive accounts, which posit that conceptual knowledge is represented in an amodal, abstract system organized by syntactic rules (Fodor, 1975). At the neural level, these theories propose that conceptual and semantic processing, including for abstract concepts, is mediated primarily by amodal semantic hubs in regions such as the temporal cortex, rather than sensorimotor areas (Visser et al., 2010; Popp et al., 2019).

Current studies suggest inconsistent conclusions stem from several factors. First, the embodied effect lacks a unified operational definition and standardized measurement. Some studies conceptualize it as a facilitatory effect, where matching activation of the body and brain helps people process information faster. However, others treat it as an inhibitory effect, in which conflicting activation of the body and brain hinders performance and slows processing (Montero-Melis et al., 2022; Shebani & Pulvermüller, 2013). Second, experimental paradigms differ across studies, contributing to inconsistent findings. Some use shallow processing tasks with minimal semantic analysis (e.g., Go/No-Go, Stroop) (X. Li et al., 2022), while others adopt deep processing tasks (e.g., sentence comprehension task, sentence plausibility judgment) (Locatelli et al., 2012; Ghavam Rankohi et al., 2025). Verbs are typically classified as concrete or abstract, yet current research faces notable limitations. Most studies examine embodied effects only within specific semantic contexts (e.g., action or concrete verbs), and cross-concept comparisons are scarce, despite evidence that concrete verbs engage motor representations (de Vega & Urrutia, 2011). Heterogeneity in abstract verbs (high vs. low motor features) complicates replication, leaving their embodied effects and influencing factors uncertain. While semantic embodiment in L1 verb processing is well established, its presence and modulators in L2 remain underexplored and inconsistent (Tian et al., 2019). Moreover, sentence-level verb processing is shaped by complex contextual factors, and whether lexical-level embodied representations generalize to sentences is unclear (Zwaan, 2021).

Collectively, existing research shows that the verb embodied effect has complex underlying mechanisms modulated by multiple factors. Thus, this study will adopt a three-level meta-analysis to categorize, integrate and evaluate individual research findings, aiming to verify the existence of the verb embodied effect and further examine whether verb features (verb concreteness, motor feature, verb layer, depth of processing task) and language status act as its potential moderators.

### 1.1. Manifestations of the Embodied Effect in Verb Processing and Learning

Following prior theoretical and empirical work (Rodríguez-Ferreiro et al., 2011; Zappa et al., 2024), verb processing refers to the cognitive operations involved in accessing, activating, integrating, and interpreting verb-related semantic representations during comprehension or production of verbs. Verb learning refers to the acquisition, encoding, retention, or strengthening of verb representations, which may involve mapping lexical forms, meanings, context, and perceptual or motor experiences. In this study, the embodied effect in verb processing refers to two aspects: (1) the influence of congruency between perceptual or motor experiences and semantic content on comprehension and processing performance, and (2) the engagement of sensorimotor representations during language processing. In verb learning, the embodied effect refers to the facilitatory or inhibitory role of sensorimotor experiences in verb (L1, L2) acquisition, typically via gestures, physical actions, movement execution, or bodily simulation. In verb processing, we hypothesize that if bodily experiences are integral to verb representation, then congruence between semantic content and perceptual actions should facilitate comprehension, whereas incongruence may impede L2 processing. For example, Secora and Emmorey (2015) examined the Action-Sentence Compatibility Effect (ACE) in American Sign Language using a semantic judgment task, testing whether perceptual processing of sign movements or verb semantics modulates the ACE. They found that motor responses were faster when the implied movement direction matched the response, indicating facilitatory under congruent conditions. In contrast, Lu and Yang (2025) observed an inhibitory effect in a verb-picture matching task. They found that sensorimotor activation was inconsistent with the task demands; participants’ cognitive performance would be impaired, and reaction times increased.

Theoretically, the embodied effect in verb learning is assumed to arise from the involvement of the sensorimotor system in lexical and conceptual representations. Enhancing sensorimotor engagement during learning is supposed to facilitate verb acquisition. Typically, studies compare embodied and non-embodied learning groups, using differences in test performance to quantify the embodied effect. For instance, X. Li et al. (2022) employed a learning-test paradigm with motor interventions for low motor feature abstract verbs. Their results showed a significant ACE in a semantic-processing syntactic classification task, with faster responses in the learning condition than the non-learning condition. Similarly, studies integrating gesture observation, imitation, and execution demonstrate that action-based learning enhances verb acquisition more effectively than traditional verbal instruction (de Nooijer et al., 2014). Although verb processing and learning examine different aspects, both address how bodily experiences influence verb comprehension and conceptual representation. The embodied effect in processing partially reveals the mechanisms of sensorimotor involvement, yet its underlying pathways remain debated, particularly regarding L1 mediation and abstract verb activation (Garello et al., 2024a; Rodríguez-Ferreiro et al., 2011). In contrast, the embodied effect in learning highlights the role of perceptual-motor experiences in linking lexical and conceptual representations. However, most studies focus on a single dimension, producing fragmented and inconsistent findings. For instance, while concrete verb semantics rely on action representations (Druks, 2002), the characteristics of embodied effects in verb learning lack systematic synthesis. Moreover, the direction of these effects (facilitatory or inhibitory) and their determinants remain contested and may coexist within the same experiment (Montero-Melis et al., 2022; Buccino et al., 2017). Accordingly, this study integrates research on embodied verb processing and learning to establish a unified framework and examine factors influencing embodied effects.

### 1.2. The Influence of Verb Features on the Embodied Effect in Verb Processing and Learning

Current inconsistencies in the embodied effect of verbs may reflect deeper theoretical disagreements regarding the nature of semantic representation. Specifically, embodied theories propose that verb meaning is grounded in sensorimotor systems, whereas amodal theories argue that verbs are represented in an amodal and abstract symbolic system. From this perspective, moderators such as verb concreteness, motor features, verb layer, and depth processing of the task are theoretically important because they are closely tied to the constitution of verb semantics. By integrating these moderators, we can test whether embodied representations are universally involved in verb processing and learning.

#### 1.2.1. Concreteness

Verb concreteness refers to the degree to which a verb denotes an action or event that is perceptually or experientially salient (Eviatar et al., 1990; Glenberg & Kaschak, 2002). Based on this dimension, verbs can be categorized into concrete verbs and abstract verbs. Concrete verbs typically denote actions or events that can be directly perceived or physically enacted (e.g., *throw*, *kick*), whereas abstract verbs more often refer to cognitive, emotional, or social processes lacking direct physical referents (e.g., *choose*, *hate*). Concreteness ratings require participants to evaluate the degree of concreteness or abstractness of words; generally, higher scores indicate greater concreteness, while lower scores indicate higher abstractness (Brysbaert et al., 2014; Paivio, 1986). A number of studies have confirmed that concrete verbs are associated with motor-embodied representations. Behavioral studies dating back to Lempert and Kinsbourne (1981) found that preschool children recalled nouns more accurately in sentences containing action verbs than in sentences with state verbs, suggesting that verb concreteness (or actionality) enhances the efficiency of verb semantic representations. More recent studies show that processing action-related language also influences brain activity. For instance, reading sentences describing manual actions, compared with abstract sentences, reduces the power of fronto-central mu rhythms, a neural marker associated with action preparation and execution. This indicates that understanding sentences about manual actions engages the sensorimotor system (Alemanno et al., 2012). In addition, X. Li et al. (2022) obtained ACE in the processing of specific verbs. This indicates that when the participants processed the individual presented two-syllable specific verbs, they mentally simulated the action and direction of movement represented by the verb. Despite extensive research, the existence of embodied representations for abstract verbs remains debated. Concrete concepts are primarily grounded in sensorimotor information, whereas abstract concepts lack physical referents and direct sensorimotor interaction, relying instead on emotional and linguistic information (Yao et al., 2016). In addition, Villani et al. (2021) used a difficulty-rating task in which participants evaluated concrete and abstract concepts while performing actions such as catching a ball, chewing gum, holding a companion’s hand, or gripping an ice pack. They found that holding hot or cold patches increased difficulty ratings for abstract concepts, suggesting that abstract concept processing can engage certain sensorimotor pathways. Conversely, other studies report no motor involvement in abstract verb processing (Innocenti et al., 2014). The inconsistencies in prior findings may reflect the heterogeneity of verbs and the common practice of treating verb concepts as homogeneous, coupled with variations and inaccuracies in material selection. Therefore, concreteness is theoretically important because it addresses a central debate in embodied cognition: whether abstract semantic representations, despite lacking direct perceptual referents, can also recruit sensorimotor grounding mechanisms.

#### 1.2.2. Motor Features

Motor features indicate how much a word’s meaning is associated with sensory or motor experiences. Words more strongly linked to bodily or movement experiences are considered to have higher motor features (Kemmerer, 2015; Van Dam & Desai, 2017). These features are typically assessed by asking participants to rate “the strength of association between a verb and action” or “the amount of movement content conveyed,” often using 5- or 7-point Likert scales. For example, X. Li et al. (2022) asked 25 participants to rate 20 abstract verbs and classified the highest- and lowest-rated verbs as high- and low-motor feature verbs (e.g., *abandon* vs. *perceive*). Only verbs showing significant differences were selected as experimental materials. High motor feature verbs typically elicit stronger engagement of motor and premotor cortical regions during comprehension, whereas low motor feature verbs rely more on higher-level semantic and conceptual processing. Motor features focus more on the sensorimotor experiences embedded in word semantics, reflecting the extent of motor-related content in concepts. Gordon and Chafetz (1990) demonstrated that children comprehend actional passives significantly better than non-actional passives, reflecting a robust actionality advantage rooted in differences in motor features and semantic engagement. Their verb-based account further emphasizes that actional events are more strongly grounded in sensorimotor experience, which aligns with the notion of high motor features in the present study. Building on this behavioral evidence, Hauk et al. (2008) suggested that the premotor and primary motor regions of the brain are organized according to different body parts (for example, hand, foot, mouth). Consequently, if these regions represent the motor features of action verbs, they should be rapidly activated during the processing of verbs related to specific actions. Supporting this neural perspective, Van Dam and Desai (2017) found that the Action-Sentence Compatibility Effect (ACE) emerged only for abstract verbs with high motor features. Similarly, Harpaintner et al. (2018) reported that the proportion of movement features in abstract words predicts activation in the left precentral and posterior central gyri. More recently, X. Li et al. (2022) further demonstrated that enhancing motor features increases the ACE during learning, accompanied by corresponding fMRI activation in typical motor areas, and that motor feature ratings predict activation magnitude. Given the heterogeneity of motor features, we hypothesize that motor features relate to the graded nature of embodied representations: verbs with richer motor information should elicit stronger embodied effects. Accordingly, this study distinguishes high- and low-motor feature verbs and further classifies them by concreteness to examine how motor features influence embodied effects.

#### 1.2.3. Verb Layer

Verb layer refers to the level of language at which a verb appears, such as a single word, a phrase, or a sentence. In this study, we distinguish three layers: single-word verbs, verb phrases, and sentence-embedded verbs (Ballard, 2022). These layers differ not only in structural complexity but also in the contextual information available during processing. Single-word verbs are processed in relative isolation, verb phrases provide local semantic associations, and sentence-level verbs are embedded within broader syntactic, semantic, and situational contexts (Mu, 2024). Against this theoretical backdrop, the transitivity hypothesis (Hopper & Thomson, 1980) well accounts for the semantic variations of verbs across different syntactic environments. High-transitivity clauses typically involve clear agents, complete actions, and rich contextual information, whereas low-transitivity clauses describe states, incomplete actions, and provide weaker context. This distinction supports the concept of verb layer: verbs embedded in larger syntactic structures are processed within a broader discourse context, which influences their semantic interpretation and engagement of sensorimotor systems. Psycholinguistic studies further indicate that processing verbs in context can enhance mental imagery and semantic representation. For example, Thibaut et al. (1995) found that the effect of action-related verbs depends on context: comprehension of action sentences is impaired when the context is incongruent, whereas non-action sentences are less affected. Stronger mental imagery improves sentence comprehension and memory, and children with better visualization abilities show superior understanding of action sentences. These findings suggest that different verb layers, reflecting varying degrees of contextual information, may differently influence embodied effects. More recently, Zwaan (2021) emphasized that examining embodied language representations requires attention to constraints such as sentence structure, context, language, text type, and genre. Context significantly influences the processing of verb-containing sentences, with the same verb activating different brain regions depending on context. For example, fMRI studies show that processing the verb “*kick*” activates motor-related regions, “*kick the ball*” elicits weaker motor activation, and the idiom “*kick the bucket*” (meaning “to die”) fails to engage motor or premotor cortices (Raposo et al., 2009). A similar effect occurs with sentences describing abstract quantity changes (Guan et al., 2013). Behavioral and neuroimaging studies demonstrate motor system involvement in processing concrete verbs (Glenberg & Kaschak, 2002; Y. Li et al., 2022; H. Wang et al., 2019). However, sentence comprehension differs from lexical processing: it is a more ecologically valid unit and involves additional factors, including contextual information that can elicit mental simulations (Barsalou, 2008; Mu, 2024). Consequently, the embodied effects of the same verb may vary across sentential contexts, and both concrete and abstract verbs exhibit altered patterns of sensorimotor engagement when processed within sentences compared with isolated lexical processing. Contextual information is theoretically relevant because it concerns whether embodied simulation is automatically triggered by isolated lexical items or dynamically constructed during contextualized language comprehension. Given these differential effects of verb layer, it is necessary to incorporate verb layer into the investigation to clarify how sentential contexts modulate the embodied processing of verbs, especially the inherent embodiment of abstract verbs that might be obscured.

#### 1.2.4. Depth of Processing Task

Depth of processing task refers to the level of cognitive processing required by an experimental task when participants engage with verbs. Tasks can range from shallow processing with minimal semantic analysis (e.g., Go/No-Go, Stroop), while others adopt deep processing tasks (e.g., sentence comprehension task, sentence plausibility judgment). Depth of processing task can effectively test whether sensorimotor activation reflects core semantic representation or later strategic elaboration during language comprehension. Previous research has shown that the recruitment of sensorimotor systems during verb processing is not automatic, but is modulated by task demands (Ostarek & Huettig, 2017; Vukovic et al., 2017). In the present study, tasks were classified according to their processing depth to examine how shallow versus deep processing tasks influence verb processing and verb learning, particularly in terms of the engagement of embodied or sensorimotor representations. Numerous task paradigms have been employed to examine the embodied effect in verb processing. Different task paradigms influence language processing and learning manifestations. For instance, Van Dam et al. (2010) investigated the embodied effect in body-related verb comprehension using a go/no-go task. Whole-brain fMRI analysis revealed a distinct neural embodied effect during action verb comprehension. Additionally, other studies have found that participants exhibited an embodied inhibitory effect in sentence plausibility judgment tasks, whereas a facilitatory effect emerged in semantic judgment tasks (Santana & de Vega, 2013; Lu & Yang, 2025). Research on verb learning has also indicated that the processing advantage of embodied learning stems from motor information activation during early verb processing, while verb semantic access relies more on deep semantic processing and extraction (Zappa et al., 2024). The inconsistencies in existing research suggest that the depth of semantic processing is also a critical factor influencing whether the sensorimotor system is activated during verb processing and learning. Accordingly, the current study classifies the depth of processing tasks across different experiments: tasks involving minimal semantic activation (e.g., Go/No-Go, Stroop) are categorized as shallow processing tasks, while tasks requiring extensive semantic activation (e.g., sentence comprehension task, sentence plausibility judgment) are classified as deep processing tasks. This classification aims to explore whether the depth of the processing task exerts an impact on the embodied effect in verb processing and learning.

### 1.3. The Impact of Language Status on the Embodied Effect of Verbs Processing and Learning

The comparison between L1 and L2 embodiment is theoretically important because it addresses whether embodied semantic representation depends on accumulated sensorimotor experience or whether semantic systems are shared across languages. Scholars have debated whether sensorimotor resources are recruited during semantic processing in both L1 and L2. Evidence shows that L1 comprehension spontaneously engages perceptual and motor information consistent with the described actions (Pulvermüller & Fadiga, 2010; Winter & Bergen, 2012). In contrast, formal L2 learning often lacks rich sensorimotor experiences, resulting in weaker perceptual and motor simulations (D. Chen et al., 2020; Norman & Peleg, 2022). For instance, X. Zhang et al. (2020) observed that Chinese-English bilinguals activated more brain regions during semantic judgment in L1 than L2. Similarly, Birba et al. (2020) found that L2 action semantics elicited longer sensorimotor activation latencies, indicating reduced sensorimotor involvement in L2 verb processing. More recently, Visani et al. (2025) combined behavioral experiments and magnetoencephalography (MEG) to examine the involvement of the motor system during L2 processing. Healthy native Italian speakers were shown hand- or foot-related actions, either as pictures or as English verbs (L2), and were asked to perform a semantic judgment task. The results showed that reaction times during L2 verb processing were significantly slower than L1 verb processing, suggesting that L2 processing involves additional processing costs. However, other studies have argued that L2 processing exhibits semantic embodiment comparable to L1, as both languages recruit similar cognitive and neural mechanisms (Ahn & Jiang, 2018; Dudschig et al., 2014). For example, Ma (2024) employed a go/no-go paradigm to test whether proficient L2 speakers would show similar motor system modulation when processing graspable verbs in L2 as in L1. The results are consistent with L1. It suggests that proficient L2 speakers engage the motor system in a similar way across languages. Additionally, limited attention has been paid to the L2 embodied effect and its potential modulators (Monaco et al., 2019; Lin et al., 2019). Furthermore, the differences in verb embodied effects between L1 and L2 remain underexplored, and the modulating factors behind such differences are still unclear. Accordingly, it is essential to systematically examine how language status shapes the embodied processing of verbs.

Taken together, the main purposes of the current study are as follows: First, to synthesize empirical studies on embodied verb processing and learning, so as to comprehensively examine the embodied effect of verbs. Second, from the perspectives of verb features and language status, to explore the impacts of verb-related factors (concreteness, motor features, verb layer, depth of processing task) and language status on the embodied effect of verb processing and learning. Meanwhile, the study attempts to address the following research questions:

(1) Does the embodied effect exist in verb processing or learning? If it does, what is the magnitude of the effect size? Is the embodied effect facilitatory or inhibitory?
(2) What are the potential moderating variables influencing the embodied effect in verb processing or learning, and is there any interaction among these variables?

## 2. Methods

The initial work of this study has been pre-registered on the OSF platform for meta-analysis (Registration https://doi.org/10.17605/OSF.IO/A7JZP).

### 2.1. Selection of Studies

We followed the PRISMA (Preferred Reporting Items for Systematic Reviews and Meta-Analyses) standards (Page et al., 2021), which are a commonly acknowledged and verified strategy for ensuring transparency and methodological rigor in systematic reviews and meta-analyses. A thorough literature search was undertaken utilizing numerous online databases, including China National Knowledge Infrastructure (CNKI), Wanfang Data, CQVIP Database, Web of Science (WOS), and ProQuest and Google Scholar. There were two main sets of selection codes for paper screening regarding: (1) search grammar concerning TS = (“verb”) AND TS = (“action” or “motion” or “sensorimotor”) AND TS = (“embodied” or “embodiment”) AND AB = (“processing” or “learning” or “acquisition”) for WOS; (2) Keywords, such as verb, embodied, embodied effect, embodied cognition, embodiment, processing, learning, and acquisition, were utilized to perform advanced searches on the titles, abstract, and keywords of the databases. The type of documents was restricted to studies concerning articles, dissertations, or theses.

### 2.2. Inclusion and Exclusion Criteria of Studies

Following the PICOS framework (Population, Intervention, Comparison, Outcomes, Study design) (Methley et al., 2014), we established rigorous inclusion and exclusion criteria for this meta-analysis. Inclusion criteria were: (1) studies published in English or Chinese; (2) empirical investigations examining the effects of embodied cognition on verb processing or learning; (3) cross-sectional or longitudinal designs with experimental (embodied) and control (non-embodied) groups, or study-test paradigms; (4) availability of sufficient effect size data, including sample size, means, standard deviations, or statistics convertible to correlation coefficients (e.g., F, t, chi-square, regression or path coefficients); and (5) participants were healthy. Exclusion criteria were: (1) interventions not aligned with the definitions of verb processing and learning or embodied effect; (2) duplicate studies or reviews; (3) experimental studies lacking either an embodied or a non-embodied control group; and (4) outcomes unrelated to verb processing or learning, or insufficient statistical information.

**Figure 1:**
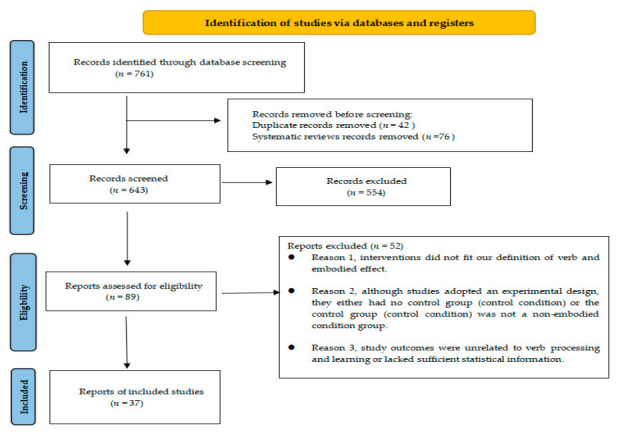
PRISMA Flow Diagram of the Inclusion and Exclusion of Studies.

This study used EndNote X9 to import literature. The selection procedure for our systematic review started on 22 August 2025, with a preliminary database search that produced 761 studies, from which 42 duplicates and 76 systematic review articles were removed. Following the initial screening of 643 studies based on abstracts and titles, 89 publications were selected for full-text review. Three coders independently assessed these articles in accordance with predefined inclusion criteria, resolving disagreements through discussion to reach consensus. Finally, 52 studies were excluded because of unavailable data or the wrong topic, and our meta-analysis, which is displayed in Figure 1, contained 37 papers. Table 1 provides major characteristics of studies included in the systematic review.

### 2.3. Coding of Study Features and Study Quality

Each study was coded in a uniform Microsoft Excel sheet, with the following parameters extracted and coded: (1) authors and year of publication; (2) number of effect size and its variance; (3) sample size; (4) verb concreteness (concrete, abstract), motion feature (high, low), verb layers (word, non-word) and depth of processing task (deep, shallow) and language status (L1, L2 and others). To guarantee accuracy and consistency in data extraction, two criteria were developed during the coding process. For studies reporting multiple samples, effect sizes were extracted for each unique sample whenever possible. Second, the method recommended by Quarmley et al. (2022), which divides the total sample size by the number of subgroups to get the size of each independent group, was used when specific subgroup sample sizes were not disclosed. Three coders carried out this coding procedure independently and meticulously recorded all pertinent details from the studies. All disagreements were resolved either through adjudication by a fourth-party researcher or by consensus following discussion. Then SPSS 25.0 was used to calculate Cohen’s kappa. The results showed that the kappa coefficient was 0.825 for study quality coding, which is almost perfect agreement. The kappa estimate ranged from 0.793 to 0.901 for five variables, demonstrating substantial to nearly perfect inter-coder reliability.

### 2.4. Three-Level Meta Analysis Process

The effect size used in this study is the standardized difference between the means of two distinct groups (Hedge’s g), a corrected form of Cohen’s d that is less impacted by small sample bias (Lipsey & Wilson, 2001). After calculating Cohen’s d based on the raw data (including sample size, mean, and standard deviation) of the embodied group and the non-embodied group in the study, Hedge’s g was computed and used as the effect size for all analyses.

The R statistical program (version 4.2.3) is used to run a three-level model, each of which has a specific function: level 1 represents sampling variation, level 2 indicates variance across effect sizes derived from the same study, and level 3 reflects variance between studies. The overall effect is modeled and calculated using the rma.mv function from the metafor package (Viechtbauer, 2010). The R program is used to implement this three-level meta-analysis in accordance with Assink and Wibbelink’s (2016) tutorial. Traditional meta-analysis cannot overcome the challenge of multiple independent effect sizes being included in a single study. Even averaging these multiple effects will lead to information loss and inaccurate estimation. However, all the effect sizes of a study can be included in the three-level model. And current meta-analyses predominantly employ the random effects model (Hedges & Vevea, 1998; Borenstein et al., 2010). The random-effects model admits that impact sizes may fluctuate between studies due to reasons other than random error, such as demographic or methodology differences. According to Cohen’s (1960) classic effect size classification criteria: small effect: Hedge’s g = 0.2; medium effect: Hedge’s g = 0.5; large effect: Hedge’s g = 0.8.

In order to control for publication bias, this study will use funnel plots, Egger’s regression, leave-one-out method and the Fail-safe *N* test to assess publication bias.

## 3. Results

### 3.1. Publication Bias

**Figure 2:**
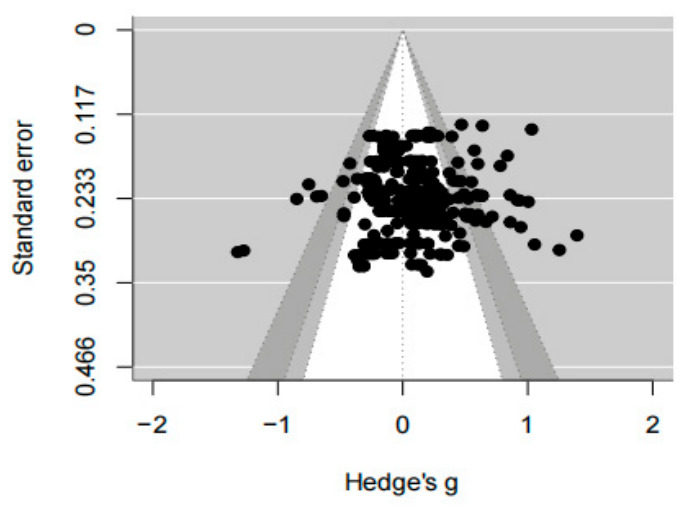
The Contour Enhanced-Funnel.

We used a contour-enhanced funnel plot to visually inspect the distribution of effect sizes and assess potential publication bias (Figure 2). Some asymmetry was observed, suggesting possible bias. Egger’s regression test for multilevel meta-analysis, which examines the relationship between standard error and effect size, was not significant (*p* = 0.070), indicating that publication bias is likely minimal (Rodgers & Pustejovsky, 2021). In addition, we conducted a comprehensive sensitivity analysis using the leave-one-out method (Mao et al., 2025) to evaluate the impact of each individual study on the overall effect size. The analysis indicated that, after removing any single study, the pooled effect size ranged between 0.22 and 0.33, demonstrating the robustness of our results. In addition, results of Fail-safe *N* test showed that *Nfs* = 4903, which exceeds Rosenthal’s (1991) criterion number of 5*N* + 10 (where *N* is the number of effect sizes included in this meta-analysis; *N* = 265). In summary, the literature included in this study exhibits publication bias, but the bias is relatively small.

### 3.2. Description of the Included Studies and Quality Assessment

This current meta-analysis included 37 studies (comprising 33 independent samples, 265 effect sizes, and 12,357 participants). Within the same study, the number of effect sizes ranged from a minimum of 1 to a maximum of 29. Among these are 12 Chinese-language publications and 25 English-language publications with a time span from 2011 to 2025. Table 1 presents major characteristics of studies included in the systematic review, and Table 2 lists the number of effect sizes for each moderator variable. Assessment of study quality revealed a mean score of 8.68 (SD = 1.19; range = 7–12), suggesting that the included literature was of overall good quality.

### 3.3. Overall Effect Sizes and Heterogeneity Analysis

**Figure 3:**
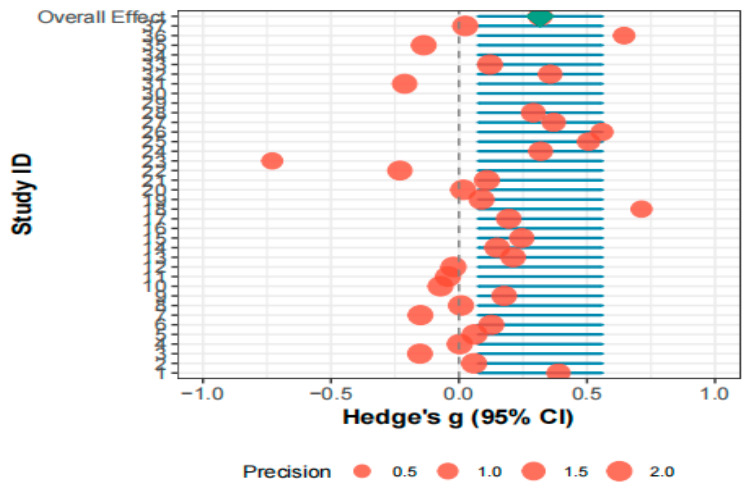
Forest Plot of Hedge’s g Effect Sizes across Studies.

The current meta-analysis employs a three-level meta-analytic model to estimate the main effect of verbs and embodied cognition. Influential case diagnostics identified five outliers. When these outliers were excluded, results (see Figure 3) from the main effects tests indicated a substantial effect size (Hedge’s g = 0. 259, *p* < 0.005, 95% CI [0.011, 0.508]) for differences between the embodied condition group and the non-embodied group, and the significant heterogeneity (Q = 914.981, *p* < 0.001, I^2^ = 71.15%). The primary analysis results remained consistent even after the removal of individual studies, indicating that the model has a high level of robustness. The sample variance (level 1), within-study variance (level 2), and variation between-study (level 3) accounted for 7.94%, 1.43%, and 90.64% of the overall variance. Both level 2 (*p* < 0.001) and level 3 (*p* < 0.001) showed significant differences according to the one-tailed log-likelihood ratio test. Based on the criteria of Higgins et al. (2003), there is low heterogeneity within studies and high heterogeneity between studies.

### 3.4. Moderator Analysis

As noted, the meta-analysis revealed substantial heterogeneity (I^2^ = 71.15%). To explore potential sources of this heterogeneity, we performed moderator analyses. The study, respectively, examined how the variables of publication year, motor feature, language status, verb concreteness, verb layers and depth of processing task moderate the overall average effect size. In addition, the current meta-analysis also focused on the interaction between verb layer and depth of processing task. These analyses suggested that differences across these moderators accounted for a portion of the observed variability.

**Figure 4:**
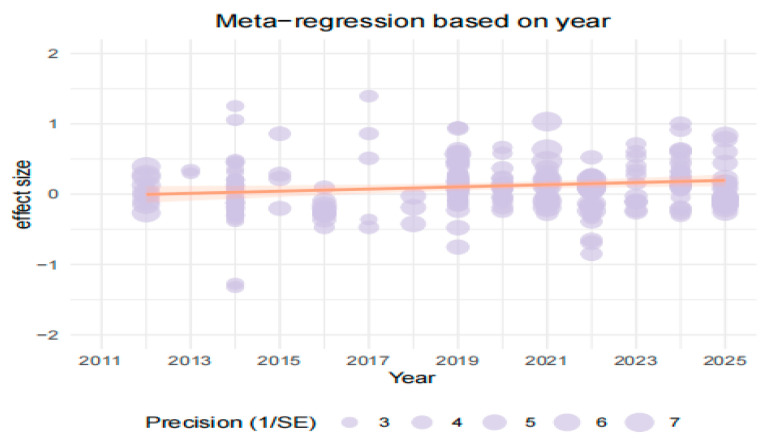
Meta-regression Based on Years.

This study adopted a mixed-effects meta-regression analysis to examine the moderating effect of publication year (a continuous variable) on the effect size, and the results (see Figure 4) showed that publication year had a significant positive moderating effect on the effect size (F(1, 263) = 5.10, *p* = 0.024).

**Figure 5:**
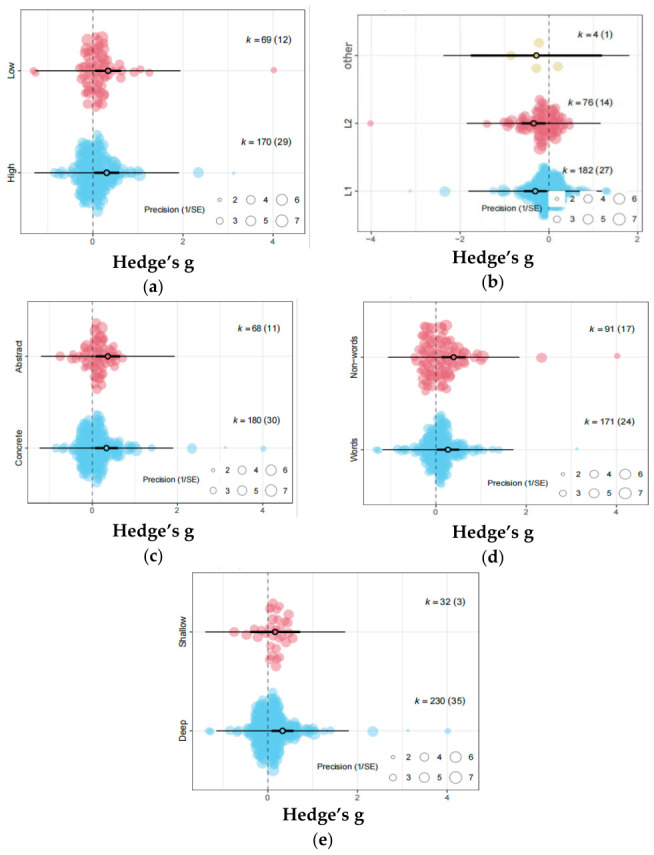
Moderating Effect of Different Moderators. (**a**) Moderating Effects of Motor Feature. *Note*: Blue = high motor feature verbs; Red = low motor feature verbs. (**b**) Moderating Effects of Language Status. *Note:* Blue = L1; Red = L2; Yellow = other languages. (**c**) Moderating Effects of Verb Concreteness. *Note*: Blue = concrete verbs; Red = abstract verbs. (**d**) Moderating Effects of Verb Layer. *Note*: Blue = word-level verbs; Red = non-word (phrase/sentence) level verbs. (**e**) Moderating Effects of Task Processing Type. *Note*: Blue = deep processing task; Red = shallow processing task. *Note*: X = effect sizes (Hedge’s g); Y = categorical factors. Each point represents an effect size (area ∝ 1/SE). *K* = number of effect sizes; number in parentheses = independent studies. Black lines = ±1 SE around the mean. Colors/symbols indicate subgroups (see legend).

The findings of the moderator studies on the connection between the target effect and verbs (see Table 2 and Figure 5) indicated significant moderating effects for verb concreteness (F(1, 246) = 2.534, *p* = 0.026) and verb layer (F(1, 260) = 4.374, *p* = 0.037), while the moderating effects of motor feature (F(1, 237) = 0.620, *p* = 0.432), language status (F(1, 260) = 0.272, *p* = 0.762) and depth of processing task (F(1, 263) = 0.480, *p* = 0.489) are not significant.

Further analysis showed that the effect of the non-word level (phrase/sentence level) was significantly stronger than that of the word level (Hedge’s g = 0.345, *p* < 0.05). while the effect of the word level on verb processing and learning was not significant (Hedge’s g = 0.188, *p* = 0.143). Also, results showed that the effect of abstract verbs was significantly stronger than that of concrete verbs (Hedge’s g = 0.315, *p* < 0.05), while the effect of concrete verbs on verb processing and learning was not significant (Hedge’s g = 0.218, *p* = 0.107).

In addition, this study examined the main effects of verb layer and depth of processing task. The results showed that the interaction moderating effect of the two variables was not significant (F(2, 262)= 2.36, *p* = 0.096). However, a significant main effect of verb layer emerged (*p* = 0.040), with non-word-level verbs showing a larger embodied effect than word-level verbs. In contrast, the main effect of the depth of processing task was not significant (*p* = 0.547), indicating no difference between shallow and deep processing tasks.

In the meta-analysis, motor feature sets High as the reference group; language status sets L1 as the reference group; verb concreteness sets concrete as the reference group; verb layer sets word as the reference group; depth of processing task sets deep as the reference group.

## 4. Discussion

The present study employed a three-level meta-analysis to systematically synthesize and analyze existing embodied cognition research on verb processing and learning, with the aim of comprehensively examining the embodied effect in these domains. An analysis of 265 effect sizes extracted from 37 included studies revealed a significant overall embodied effect of verb processing and learning (Hedges’ g = 0.259), indicating the presence of an embodied effect in verb processing and learning, and that the activation of the sensorimotor system can modulate verb comprehension and conceptual representation. Subgroup analysis results demonstrated that verb concreteness and verb layer exerted significant moderating effects on the embodied effect of verb processing and learning, whereas the moderating roles of motor features, depth of processing task, and language status were not statistically significant. Also, the interaction effect of the verb layer and the depth of processing task was not significant.

### 4.1. The Impact of Verb Features on the Embodied Effect in Verb Processing and Learning

#### 4.1.1. Concreteness

This study compared whether there were differences in embodied effects between concrete verbs and abstract verbs. The results demonstrated that the embodied effect of verbs is modulated by verb concreteness. Specifically, the magnitude of the embodied effect was greater for abstract verbs than for concrete verbs, which is inconsistent with the conclusions of previous studies. Although some individual studies (e.g., X. Li et al., 2022) reported Action-Sentence Compatibility Effect (ACE) during concrete verb processing, the present meta-analysis synthesized evidence across a broader range of paradigms and materials, revealing that abstract verbs overall showed relatively stronger embodied effects. The current study revealed a counterintuitive phenomenon, that is, abstract verbs exhibited a larger magnitude of embodied effect during processing. This finding challenged traditional cognitive views, which assume that all conceptual representations, including abstract concepts, are independent of sensorimotor experience and are encoded as amodal symbolic structures (Amodal Theories; Fodor, 1975). We propose three potential explanations for this phenomenon. On the one hand, the semantics of abstract verbs lack intuitive sensorimotor anchors, which necessitate the recruitment of more complex metaphorical embodied simulations (e.g., using the bodily experience of “upward movement” to represent abstract meanings such as “positivity” or “improvement”; Lakoff & Johnson, 1980). Such simulations involve the integration of broader sensorimotor systems, which may elicit stronger embodied effects. On the other hand, the sensorimotor features of concrete verbs are direct and fixed (e.g., the verb “*kick*” corresponds to leg movements), confining their embodied representations to specific body regions or movement patterns with limited scope and intensity activation. In contrast, the embodied representations of abstract verbs are indirect and multidimensional, requiring the integration of information across multiple domains of bodily experience to achieve semantic anchoring. Furthermore, previous neurocognitive studies have suggested that the embodied representations of abstract concepts may involve broader multimodal semantic integration, including motor, emotional, and spatial systems (e.g., Liang, 2018; Liu, 2020; Yu & Lu, 2021). From this perspective, the relatively stronger embodied effects observed for abstract verbs in the present meta-analysis may reflect the greater reliance of abstract semantic processing on distributed bodily and experiential information. However, because the current meta-analysis primarily synthesized behavioral evidence, such neurocognitive interpretations should be considered tentative rather than direct conclusions supported by the present data.

#### 4.1.2. Motor Features

The current study examined whether embodied effects differed between verbs with low and high motor features. Results showed no significant differences, suggesting that motor features largely do not modulate the embodied effect, which appears relatively consistent across verbs with varying motor features. The lack of a significant effect of motor features can be understood from the perspective of the amodal semantic hub theory of the anterior temporal lobes (ATLs). According to a meta-analysis by Visser et al. (2010), the bilateral ATLs serve as an amodal semantic hub, supporting conceptual processing across different sensory modalities and types of stimuli. From this view, semantic representations are organized within a shared conceptual system, rather than relying mainly on modality-specific motor information. Therefore, verbs with high and low motor features may engage similar semantic processing mechanisms, leading to comparable embodied effects. However, previous studies have argued that verbs with high motor features have higher imageability than low motor features, making it easier to construct complete action simulations in the brain and drive precise motor planning and action parameter representations, thus leading to significantly greater activation (Van Dam et al., 2010; Jin & Li, 2022). Similarly, Kemmerer (2015) found that verbs with high motor features specifically activate the precentral motor cortex, and this activation exhibits somatotopy (i.e., leg/hand/oral action verbs correspondingly activate the relevant somatic motor regions); in contrast, verbs with low motor features generally do not spontaneously activate the precentral motor cortex. However, the findings of the current study revealed that identical embodied effects were observed during the processing of verbs with high versus low motor features. Given that the experimental materials of the included literature in this study covered both concrete and abstract verbs, there were cases where the evaluation criteria for some materials were ambiguous, making it difficult to define whether the target verbs belonged to the category of high motor feature or low motor feature verbs clearly. Additionally, the number of low motor feature verbs in the included literature was relatively small. These factors may affect the results of the subgroup analysis to some extent.

#### 4.1.3. Verb Layer

The present meta-analysis compared embodied effects across three linguistic layers: single verbs, verb phrases, and verbs embedded in sentences. The results demonstrated that the verb layer significantly moderated the magnitude of embodied effects, with stronger effects observed for phrases and sentence-level verbs than for isolated single-word verbs. At the meta-analytic level, these findings support the view that contextual embedding facilitates the recruitment of embodied or sensorimotor representations during verb processing. Previous individual studies have reported different activation patterns across linguistic levels (e.g., Raposo et al., 2009). Such inconsistencies may reflect methodological heterogeneity across studies, including differences in task design, stimulus selection, and analytical approaches. Importantly, verb processing in sentences is shaped by multiple contextual factors, including sentential context, tense, and perspective. Context may also alter the semantic properties of verbs. For example, concrete verbs used in metaphorical contexts no longer denote literal actions, and their association with the sensorimotor system may consequently be modified (Kacinik, 2014; Mu, 2024; H. Wang et al., 2019; Y. Feng & Zhou, 2019, 2021). Across the broader body of evidence synthesized in the present meta-analysis, stronger embodied effects emerged under richer contextual conditions. These findings suggest that phrases and sentences offer supportive semantic contexts, which facilitate embodied simulation (Barsalou, 2008). In turn, this support strengthens embodied effects during verb processing and learning.

#### 4.1.4. Depth of Processing Task

Studies have found that both deep and shallow processing tasks exhibit significant embodied effects of verbs, yet there was no significant difference in the magnitude of the embodied effect in different processing depths. It indicates that an observable embodied effect exists in verb processing and learning. This pattern aligns with a body of literature indicating that sensorimotor engagement during language processing is flexible and sensitive to task constraints (Ostarek & Huettig, 2017; Vukovic et al., 2017). Although some individual studies have reported that embodied effects emerge primarily under deep semantic processing conditions, the present meta-analysis did not identify a significant moderating effect of depth of processing task. Specifically, X. Li et al. (2022) found that enhancing the motor features of words increased motor-system involvement in abstract verb processing across semantic tasks, such as part-of-speech judgment, word-meaning matching, and pseudoword judgment. In contrast, no Action–Sentence Compatibility Effect (ACE) was observed in a word-color judgment task that did not require semantic processing. Based on these findings, the authors suggested that the emergence of embodied effects in abstract verb processing may depend on the extent of semantic extraction required by the task. However, the current meta-analysis synthesizes a substantially broader body of evidence and found that the depth of processing task appears to exert a relatively weak moderating role overall. According to Dual Coding Theory, the imagery system (or sensorimotor system) represents surface-level sensorimotor features of concepts, such as shape, color, and motor attributes, whereas the verbal system encodes lexical and syntactic information (Paivio, 1986; Liu, 2020; D. Chen et al., 2020). Concrete concepts engage both systems, while abstract concepts are primarily represented in the verbal system. The linguistic system dominates abstract verb processing, but the sensorimotor system can be recruited during deep semantic processing, depending on context. Nevertheless, evidence shows that tasks involving sensorimotor information, even for surface features, inevitably engage the full semantic and syntactic representations of both concrete and abstract verbs.

### 4.2. The Impact of Language Status on the Embodied Effect in Verbs Processing and Learning

After incorporating language status as a moderating variable into the analysis, the current study found that the moderating effect of language status was not statistically significant. Therefore, the present findings do not provide sufficient evidence to conclude that language status significantly moderates embodied effects in verb processing and learning. Although the moderator analysis did not reveal a statistically significant difference between the two language conditions, the embodied effect size was descriptively larger in L1 (Hedges’ g = 0.275) than in L2 (Hedges’ g = 0.222). This finding is generally consistent with the conclusions of previous research (Y. Zhang et al., 2024; Winter & Bergen, 2012). A number of previous studies has demonstrated that semantic comprehension in L1 spontaneously evokes perceptual and motor information that aligns with the perceptions and actions described by language (Glenberg & Kaschak, 2002; Pulvermüller & Fadiga, 2010). Comparing with L1 learning, L2 learning typically lacks the rich sensorimotor experiences inherent to native language learning. As a result, L2 comprehension processes are deficient in robust perceptual and motor simulation (D. Chen et al., 2020; Norman & Peleg, 2022). Although some individual studies have argued that L1 and L2 recruit different cognitive and neural mechanisms during embodied semantic processing (Ahn & Jiang, 2018; Dudschig et al., 2014), the present meta-analytic findings suggest that these differences may be only partial, as no statistically significant difference was observed in the overall magnitude of embodied effects across language status. The current study proposed that a plausible explanation is that processing L1 verbs more easily activates sensorimotor brain regions, which can modulate task performance. By contrast, L2 verb processing may be influenced by participants’ L2 proficiency. Previous research has suggested that less proficient bilinguals allocate more cognitive resources to L2 processing, resulting in insufficient resources to activate the corresponding sensorimotor systems (Keysar et al., 2012). Another potential interpretation is that, compared with the refined and profound representations of L1 verbs, L2 verb representations are less elaborate and deep-seated. This is because L2 verb representations lack the sensorimotor experiences that link L2 linguistic symbols to their referential concepts. After synthesizing previous experimental findings and leveraging a sufficiently large sample size, the current study found that both L1 and L2 exhibit verb embodied effects, but there is no significant difference in these effects. Overall, the effect size for L1 was slightly larger than that for L2. It also implies that enhancing learners’ sensorimotor experiences during L2 verb learning might facilitate the establishment of connections between L2 linguistic symbols and their corresponding conceptual representations, thereby promoting L2 verb learning.

### 4.3. Limitations and Future Studies

By synthesizing research on embodied cognition in verb processing and learning, and influential moderators such as verb features and language status, this study examined the characteristics, manifestations, and influencing factors of verb embodied effects. The findings have several implications for future research. First, the three-level meta-analysis confirms the existence of the embodied effect, helping to resolve prior controversies and providing a foundation for further investigation of its cognitive and functional mechanisms. Second, abstract verbs exhibited stronger embodied effects than concrete verbs, suggesting that verb concreteness warrants greater attention in future studies. Finally, embodied effects were more pronounced for verb phrases and sentences than for isolated verbs, highlighting the importance of verb layer in facilitating conceptual understanding.

Despite its contributions, this study has several limitations. First, subgroup sample sizes were uneven in some analyses, which may reduce the stability of observed moderating effects. For instance, the number of isolated verbs, verb phrases, and sentences was unbalanced, and verb phrases were combined with sentences due to insufficient data. Few studies have examined the competitive activation of sensorimotor and linguistic representations during verb processing, limiting relevant empirical evidence. Second, only behavioral data were included, without neurophysiological or neuroimaging evidence to clarify the neural mechanisms of verb embodiment. Third, most participants were university students, restricting generalizability to broader populations (Zhu, 2017; S. R. Zhang, 2021). Additionally, potential publication bias was suggested by funnel plots and statistical tests, indicating that unpublished null or negative findings may be missing. Substantial variability in experimental paradigms (e.g., ACE, Stroop, lexical decision, and sentence verification tasks) introduced procedural heterogeneity that could influence the magnitude of embodied effects. Cross-linguistic differences among Chinese, English, Spanish, Italian, and German, which involve distinct typological and orthographic properties, were not formally examined as moderators, nor were differences between L1 and L2 embodiment effects. Given that language proficiency may moderate embodiment in bilinguals, its omission represents an important limitation. Future research should adopt balanced designs, integrate neuroimaging data, recruit diverse populations, control for cross-linguistic factors, include unpublished studies, and use consistent experimental paradigms to enhance the reliability and replicability of verb embodiment research.

## 5. Conclusions

The present study provides evidence that embodied representations influence verb processing and learning. Effect sizes vary across studies, reflecting substantial heterogeneity, and are moderated by verb concreteness and verb layer. Specifically, abstract verbs tend to show stronger embodied effects than concrete verbs, and non-word-level (phrases and sentences) verbs generally elicit more pronounced effects than isolated verbs. Overall, the findings support a general role of embodied representations in verb processing and learning, while highlighting that the magnitude and consistency of these effects may depend on verb concreteness and contextual information provided by different verb layers. Taken together, these findings have important implications for L2 verb instruction, suggesting that incorporating contextual and embodied experiences may enhance verb processing and learning.

## Figures and Tables

**Table 1:** Major characteristics of studies included in the systematic review.

Study & Year	*N*	Procedure	Stimuli	Dependent Variable
Y. Feng and Zhou (2019)	75	Spatial cueing paradigm	Verbs (concrete/abstract)	Reaction time (RT)
Y. Feng and Zhou (2021)	92	Picture-priming paradigm	Verb phrases/sentences	RT, accuracy
de Vega and Urrutia (2011)	172	ACE paradigm	Action sentences	RT
Muraki et al. (2022)	36	Go/No-go syntactic categorization	Abstract verbs	Accuracy
Muraki et al. (2022)	61	Syntactic judgment/recognition	Abstract verbs	RT, accuracy
Mu (2024)	35	ACE paradigm	Abstract verb sentences	RT, accuracy
Zhao (2022)	39	SPT/VT encoding task	Verb phrases	Recognition accuracy
Dalla Volta et al. (2014)	26	Verb categorization task	Body-related verbs	RT, accuracy
X. Li et al. (2022)	255	ACE paradigm	Concrete/abstract verbs	RT
Sun (2022)	25	Word recognition task	Verbs/phrases	Recognition RT
Yu and Lu (2021)	38	Phrase priming task	Concrete/abstract verbs	Lexical decision RT
Yu and Lu (2021)	100	Word learning task	Abstract verbs	RT, accuracy
Ma (2024)	56	Vertical Stroop task	Approach/avoidance verbs	RT, accuracy
Rolán et al. (2023)	30	Verb identification task	Action verbs	RT
Liu (2020)	42	Lexical decision task	Body action verbs	RT
H. Wang et al. (2019)	18	Sentence plausibility judgment	Action verb sentences	Acceptance rate
S. R. Zhang (2021)	120	Metaphor comprehension task	Verb phrases	RT
M. Chen (2020)	37	Lexical decision with context	Dynamic verbs	RT, accuracy
L. L. Li (2019)	32	Lexical decision task	Hand/foot verbs	RT
B. Wang et al. (2019)	118	Spatial direction judgment	Vertical spatial verbs	RT, accuracy
Sidhu and Pexman (2016)	56	Verb recognition task	High/low embodied verbs	Hit rate, false alarm rate
Zhong (2022)	40	Action learning task	Novel verbs	Recall accuracy
Santana and de Vega (2013)	25	Sentence judgment task	Action sentences	RT, accuracy
J. Li (2023)	32	Syntactic judgment task	Mental/emotional verbs	RT, accuracy
Lin et al. (2019)	30	Syntactic classification task	High/low embodied verbs	RT
Zhu (2017)	31	Lexical decision/fill-in-the-blank	Motion verbs	RT, accuracy
Secora and Emmorey (2015)	42	Sentence sense judgment	ASL action phrases	RT
H. Wang et al. (2024)	102	Action priming task	Verb metaphors	RT
Lu and Yang (2025)	61	Verb-picture matching task	Action verbs	RT, accuracy
Liang (2018)	60	Gesture learning task	Action verbs	Recognition accuracy
J. Feng (2020)	30	Syntactic classification task	Contextual verbs	RT
Locatelli et al. (2012)	19	Action training task	Hand-related sentences	RT, accuracy
Garello et al. (2024a)	40	Action video priming task	Literal/metaphorical sentences	RT
Rolán et al. (2023)	41	Typing task	Action verbs	First/word latency
Garello et al. (2024b)	40	L1/L2 sentence judgment task	L1/L2 action sentences	RT
Ghavam Rankohi et al. (2025)	344	Typing task	Manual/non-manual verbs	RT, accuracy
de Nooijer et al. (2014)	48	Gesture observation/imitation learning task	concrete/abstract verbs	Recall accuracy, Recognition RT

**Table 2:** The moderating effect of the relationship between verbs and the embodied effect.

Moderators	*k*	Hedge’s g (95% CI)	*β* (95% CI)	*F*	*p*	Variance (Level 2)	Variance (Level 3)
**Motor feature**				0.620	0.432	0.007 ***	0.627 ***
High	170	0.249 [−0.034, 0.532]					
Low	69	0.289 [−0.002, 0.579]	0.040 [−0.060, 0.139]				
**Language Status**				0.272	0.762	0.009 ***	0.565 ***
L1	182	0.275 [0.014, 0.535]					
L2	76	0.222 [−0.056, 0.500]	0.053 [−0.089, 0.194]				
ASL	4	0.225 [−1.272, 1.723]	0.049 [−1.469, 1.568]				
**Verb concreteness**				2.534	0.026	0.009 ***	0.521 ***
Concrete	180	0.218 [0.011, 0.425]					
Abstract	68	0.315 [0.097, 0.533]	−0.270 [−0.437,−0.102]				
**Verb Layer**				4.374	0.037	0.009 ***	0.514 ***
Word	171	0.188 [−0.064, 0.440]					
Non-word	91	0.345 [0.009, 0.681]	−0.130 [−0.605, 0.345]				
**Depth of Processing Task**				0.480	0.489	0.008 ***	0.540 ***
Deep	233	0.270 [0.019,0.521]					
Shallow	32	0.100 [−0.417. 0.617]	−0.170 [−0.653.0.313]				

Note: *k* = number of effect sizes; CI = confidence interval; *β* = estimated regression coefficient; Variance (Level 2) = variance between effect sizes extracted from the same study; Variance (Level 3) = variance between studies; *** *p* < 0.001.

## Data Availability

The original contributions presented in this study are included in the article/Supplementary Materials. Further inquiries can be directed to the corresponding author.

## Supplementary Materials

The following supporting information can be downloaded at: https://www.mdpi.com/article/10.3390/bs16060914/s1, PRISMA 2020 checklist.

## References

[B1-behavsci-16-00914] Ahn S., Jiang N. (2018). Automatic semantic integration during L2 sentential reading. Bilingualism: Language and Cognition.

[B2-behavsci-16-00914] Alemanno F., Houdayer E., Cursi M., Velikova S., Tettamanti M., Comi G., Cappa S. F., Leocani L. (2012). Action-related semantic content and negation polarity modulate motor areas during sentence reading: An event-related desynchronization study. Brain Research.

[B3-behavsci-16-00914] Assink M., Wibbelink C. J. (2016). Fitting three-level meta-analytic models in R: A step-by-step tutorial. Quantitative Methods for Psychology.

[B4-behavsci-16-00914] Ballard K. (2022). The frameworks of English: Introducing language structures.

[B5-behavsci-16-00914] Barsalou L. W. (2008). Grounded cognition. Annual Review of Psychology.

[B6-behavsci-16-00914] Birba A., Beltrán D., Caro M. M., Trevisan P., Kogan B., Sedeño L., Ibáñez A., García A. M. (2020). Motor-system dynamics during naturalistic reading of action narratives in first and second language. NeuroImage.

[B7-behavsci-16-00914] Borenstein M., Hedges L. V., Higgins J. P., Rothstein H. R. (2010). A basic introduction to fixed-effect and random-effects models for meta-analysis. Research Synthesis Methods.

[B8-behavsci-16-00914] Brysbaert M., Warriner A., Kuperman V. (2014). Concreteness ratings for 40 thousand generally known English word lemmas. Behavior Research Methods.

[B9-behavsci-16-00914] Buccino G., Marino B. F., Bulgarelli C., Mezzadri M. (2017). Fluent speakers of a second language process graspable nouns expressed in L2 like in their native language. Frontiers in Psychology.

[B10-behavsci-16-00914] Chen D., Wang R., Zhang J., Liu C. (2020). Perceptual representations in L1, L2 and L3 comprehension: Delayed sentence–picture verification. Journal of Psycholinguistic Research.

[B11-behavsci-16-00914] Chen M. (2020). An embodied cognitive study on the processing of dynamic motion verbs among native Chinese speakers. Master’s thesis.

[B12-behavsci-16-00914] Cohen J. (1960). A coefficient of agreement for nominal scales. Educational and Psychological Measurement.

[B13-behavsci-16-00914] Dalla Volta R., Fabbri-Destro M., Gentilucci M., Avanzini P. (2014). Spatiotemporal dynamics during processing of abstract and concrete verbs: An ERP study. Neuropsychologia.

[B14-behavsci-16-00914] de Nooijer J. A., van Gog T., Paas F., Zwaan R. A. (2014). Words in action: Using gestures to improve verb learning in primary school children. Gesture.

[B15-behavsci-16-00914] de Vega M., Urrutia M. (2011). Counterfactual sentences activate embodied meaning: An action–sentence compatibility effect study. Journal of Cognitive Psychology.

[B16-behavsci-16-00914] Druks J. (2002). Verbs and nouns—A review of the literature. Journal of Neurolinguistics.

[B17-behavsci-16-00914] Dudschig C., de la Vega I., Kaup B. (2014). Embodiment and secondlanguage: Automatic activation of motor responses during processing spatially associated L2 words and emotion L2 words in a vertical Stroop paradigm. Brain and Language.

[B18-behavsci-16-00914] Eviatar Z., Menn L., Zaidel E. (1990). Concreteness: Nouns, verbs, and hemispheres. Cortex.

[B19-behavsci-16-00914] Feng J. (2020). The syntactic contextual constraints on the embodied effect of L2 verbs. Master’s thesis.

[B20-behavsci-16-00914] Feng Y., Zhou R. (2019). The embodied cognitive effect of L2 verb processing under the spatial cueing paradigm. Foreign Language Research.

[B21-behavsci-16-00914] Feng Y., Zhou R. (2021). Does embodiment of verbs influence predicate metaphor processing in a second language? Evidence from picture priming. Frontiers in Psychology.

[B22-behavsci-16-00914] Fodor J. A. (1975). The language of thought.

[B23-behavsci-16-00914] Garello S., Ferroni F., Gallese V., Ardizzi M., Cuccio V. (2024a). The role of embodied cognition in action language comprehension in L1 and L2. Scientific Reports.

[B24-behavsci-16-00914] Garello S., Ferroni F., Gallese V., Cuccio V., Ardizzi M. (2024b). From breaking bread to breaking hearts: Embodied simulation and action language comprehension. Language, Cognition and Neuroscience.

[B25-behavsci-16-00914] Ghavam Rankohi Z., Liepelt R., Luchterhand-Dehn J., Sperl L. (2025). Embodied cognition in native and foreign language–evidence from a typing task. Journal of Cognitive Psychology.

[B26-behavsci-16-00914] Glenberg A. M., Kaschak M. P. (2002). Grounding language in action. Psychonomic Bulletin & Review.

[B27-behavsci-16-00914] Gordon P., Chafetz J. (1990). Verb-based versus class-based accounts of actionality effects in children’s comprehension of passives. Cognition.

[B28-behavsci-16-00914] Guan C. Q., Meng W., Yao R., Glenberg A. M. (2013). The motor system contributes to comprehension of abstract language. PLoS ONE.

[B29-behavsci-16-00914] Harpaintner M., Trumpp N. M., Kiefer M. (2018). The semantic content of abstract concepts: A property listing study of 296 abstract words. Frontiers in Psychology.

[B30-behavsci-16-00914] Hauk O., Shtyrov Y., Pulvermüller F. (2008). The time course of action and action-word comprehension in the human brain as revealed by neurophysiology. Journal of Physiology-Paris.

[B31-behavsci-16-00914] Hedges L. V., Vevea J. L. (1998). Fixed-and random-effects models in meta-analysis. Psychological Methods.

[B32-behavsci-16-00914] Higgins J. P. T., Thompson S. G., Deeks J. J., Altman D. G. (2003). Measuring inconsistency in meta-analyses. BMJ: British Medical Journal.

[B33-behavsci-16-00914] Hopper P., Thomson S. A. (1980). Transitivity in grammar and discourse. Language.

[B34-behavsci-16-00914] Innocenti A., De Stefani E., Sestito M., Gentilucci M. (2014). Understanding of action-related and abstract verbs in comparison: A behavioral and TMS study. Cognitive Processing.

[B35-behavsci-16-00914] Jin H., Li X. (2022). Embodied representation of abstract verbs: The influence of motor features. Psychological Science.

[B36-behavsci-16-00914] Kacinik N. (2014). Sticking your neck out and burying the hatchet: What idioms reveal about embodied simulation. Frontiers in Human Neuroscience.

[B37-behavsci-16-00914] Kemmerer D. (2015). Are the motor features of verb meanings represented in the precentral motor cortices? Yes, but within the context of a flexible, multilevel architecture for conceptual knowledge. Psychonomic Bulletin & Review.

[B38-behavsci-16-00914] Keysar B., Hayakawa S. L., An S. G. (2012). The foreign-language effect: Thinking in a foreign tongue reduces decision biases. Psychological Science.

[B39-behavsci-16-00914] Lakoff G., Johnson M. (1980). Metaphors we live by.

[B40-behavsci-16-00914] Lempert H., Kinsbourne M. (1981). How young children represent sentences: Evidence from the superiority of noun recall from action as compared to stative sequences. Journal of Psycholinguistic Research.

[B41-behavsci-16-00914] Li J. (2023). A study on the processing differences between bodily and non-bodily stative abstract verbs among Chinese EFL learners. Master’s thesis.

[B42-behavsci-16-00914] Li L. L. (2019). A comparative study on the embodied semantic processing effects of Chinese hand-foot verbs. Master’s thesis.

[B43-behavsci-16-00914] Li X., Luo D., Wang C., Xia Y., Jin H. (2022). Motor features of abstract verbs determine their representations in the motor system. Frontiers in Psychology.

[B44-behavsci-16-00914] Li Y., Lu X., Wang Y., Wang H., Wang Y. (2022). Is the processing of Chinese verbal metaphors simulated or abstracted? Evidence from an ERP study. Frontiers in Psychology.

[B45-behavsci-16-00914] Liang M. X. (2018). The influence of gestures on verb processing: From the perspective of embodied cognition. Master’s thesis.

[B46-behavsci-16-00914] Lin L. H., Hu C. Q., Yu S. Z. (2019). An empirical study on the relationship between verb semantic processing and verb embodiment among Chinese English learners. Foreign Language Teaching and Research.

[B47-behavsci-16-00914] Lipsey W., Wilson B. (2001). Practical meta-analysis..

[B48-behavsci-16-00914] Liu Y. X. (2020). An embodied study on the semantic comprehension of Chinese monosyllabic verbs. Master’s thesis.

[B49-behavsci-16-00914] Locatelli M., Gatti R., Tettamanti M. (2012). Training of manual actions improves language understanding of semantically related action sentences. Frontiers in Psychology.

[B50-behavsci-16-00914] Lu X., Yang J. (2025). Second language embodiment of action verbs: The impact of bilingual experience as a multidimensional spectrum. Bilingualism: Language and Cognition.

[B51-behavsci-16-00914] Ma K. X. (2024). A study on the cognitive neural mechanisms of embodied representation of L2 verbs in Chinese-English bilinguals. Master’s thesis.

[B52-behavsci-16-00914] Mao Y., Shen Y., Yang Q., Shi Q., Li S. (2025). A leave-one-out algorithm for contribution analysis in component network meta-analysis. BMC Medical Research Methodology.

[B53-behavsci-16-00914] Methley A. M., Campbell S., Chew-Graham C., McNally R., Cheraghi-Sohi S. (2014). PICO, PICOS and SPIDER: A comparison study of specificity and sensitivity in three search tools for qualitative systematic reviews. BMC Health Services Research.

[B54-behavsci-16-00914] Monaco E., Jost L. B., Gygax P. M., Annoni J.-M. (2019). Embodied semantics in a second language: Critical review and clinical implications. Frontiers in Human Neuroscience.

[B55-behavsci-16-00914] Montero-Melis G., van Paridon J., Ostarek M., Bylund E. (2022). No evidence for embodiment: The motor system is not needed to keep action verbs in working memory. Cortex.

[B56-behavsci-16-00914] Mu L. (2024). Embodied motor representations of abstract verb sentences. Master’s thesis.

[B57-behavsci-16-00914] Muraki E. J., Sidhu D. M., Pexman P. M. (2022). Heterogenous abstract concepts: Is “ponder” different from “dissolve”?. Psychological Research.

[B58-behavsci-16-00914] Norman T., Peleg O. (2022). The reduced embodiment of a second language. Bilingualism: Language and Cognition.

[B59-behavsci-16-00914] Ostarek M., Huettig F. (2017). A task-dependent causal role for low-level visual processes in spoken word comprehension. Journal of Experimental Psychology: Learning, Memory, and Cognition.

[B60-behavsci-16-00914] Page M. J., McKenzie J. E., Bossuyt P. M., Boutron I., Hoffmann T. C., Mulrow C. D., Moher D. (2021). The PRISMA 2020 statement: An updated guideline for reporting systematic reviews. British Medical Journal.

[B61-behavsci-16-00914] Paivio A. (1986). Mental representations: A dual coding approach.

[B62-behavsci-16-00914] Popp M., Trumpp N. M., Sim E. J., Kiefer M. (2019). Brain activation during conceptual processing of action and sound verbs. Advances in Cognitive Psychology.

[B63-behavsci-16-00914] Postle N., McMahon K. L., Ashton R., Meredith M., de Zubicaray G. I. (2008). Action word meaning representations in cytoarchitectonically defined primary and premotor cortices. Neuroimage.

[B64-behavsci-16-00914] Pulvermüller F., Fadiga L. (2010). Active perception: Sensorimotor circuits as a cortical basis for language. Nature Reviews Neuroscience.

[B65-behavsci-16-00914] Quarmley M., Feldman J., Grossman H., Clarkson T., Moyer A., Jarcho M. (2022). Testing effects of social rejection on aggressive and prosocial behavior: A meta-analysis. Aggressive Behavior.

[B66-behavsci-16-00914] Raposo A., Moss H. E., Stamatakis E. A., Tyler L. K. (2009). Modulation of motor and premotor cortices by actions, action words and action sentences. Neuropsychologia.

[B67-behavsci-16-00914] Rodgers M. A., Pustejovsky J. E. (2021). Evaluating meta-analytic methods to detect selective reporting in the presence of dependent effect sizes. Psychological Methods.

[B68-behavsci-16-00914] Rodríguez-Ferreiro J., Gennari S. P., Davies R., Cuetos F. (2011). Neural correlates of abstract verb processing. Cognition Neuroscience.

[B69-behavsci-16-00914] Rolán K., Sánchez-Borges I., Kogan B., García-Marco E., Álvarez C. J., de Vega M., García A. M. (2023). The embodied typist: Bimanual actions are modulated by words’ implied motility and number of evoked limbs. PLoS ONE.

[B70-behavsci-16-00914] Rosenthal R. (1991). Meta-analytic procedures for social research.

[B71-behavsci-16-00914] Santana E. J., de Vega M. (2013). An ERP study of motor compatibility effects in action language. Brain Research.

[B72-behavsci-16-00914] Secora K., Emmorey K. (2015). The action-sentence compatibility effect in ASL: The role of semantics vs. perception. Language and Cognition.

[B73-behavsci-16-00914] Shebani Z., Pulvermüller F. (2013). Moving the hands and feet specifically impairs working memory for arm- and leg-related action words. Cortex.

[B74-behavsci-16-00914] Sidhu D. M., Pexman P. M. (2016). Is moving more memorable than proving? Effects of embodiment and imagined enactment on verb memory. Frontiers in Psychology.

[B75-behavsci-16-00914] Sun H. L. (2022). The influence of verbs and contextual diversity on the operational effect of action memory. Master’s thesis.

[B76-behavsci-16-00914] Thibaut J. P., Rondal J. A., KÄens A. M. (1995). Actionality and mental imagery in children’s comprehension of declaratives. Journal of Child Language.

[B77-behavsci-16-00914] Tian L., Chen H., Zhao W., Wu J., Zhang Q., De A., Leppänen P., Cong F., Parviainen T. (2019). The role of motor system in action-related language comprehension in L1 and L2: An fMRI study. Brain and Language.

[B78-behavsci-16-00914] Van Dam W. O., Desai R. H. (2017). Embodied simulations are modulated by sentential perspective. Cognitive Science.

[B79-behavsci-16-00914] Van Dam W. O., Rueschemeyer S. A., Bekkering H. (2010). How specifically are action verbs represented in the neural motor system: An fMRI study. Neuroimage.

[B80-behavsci-16-00914] Viechtbauer W. (2010). Conducting meta-analyses in R with the metafor package. Journal of Statistical Software.

[B81-behavsci-16-00914] Villani C., Lugli L., Liuzza M. T., Nicoletti R., Borghi A. M. (2021). Sensorimotor and interoceptive dimensions in concrete and abstract concepts. Journal of Memory and Language.

[B82-behavsci-16-00914] Visani E., Rossi Sebastiano D., Garofalo G., Duran D., Tangorra M., Mezzadri M., Buccino G. (2025). Processing L2 action verbs shares the same mechanisms for processing L1 items: Evidence from a combined behavioral and MEG study. Frontiers in Psychology.

[B83-behavsci-16-00914] Visser M., Jefferies E., Lambon Ralph M. A. (2010). Semantic processing in the anterior temporal lobes: A meta-analysis of the functional neuroimaging literature. Journal of Cognitive Neuroscience.

[B84-behavsci-16-00914] Vukovic N., Feurra M., Shpektor A., Myachykov A., Shtyrov Y. (2017). Primary motor cortex functionally contributes to language comprehension: An online rTMS study. Neuropsychologia.

[B85-behavsci-16-00914] Wang B., Li Z. R., Wu L. M., Zhang J. J. (2019). The role of embodied simulation in the comprehension of Chinese limb-action verbs. Acta Psychologica Sinica.

[B86-behavsci-16-00914] Wang H., Li J., Wang X., Jiang M., Cong F., de Vega M. (2019). Embodiment effect on the comprehension of Mandarin manual action language: An ERP study. Journal of Psycholinguistic Research.

[B87-behavsci-16-00914] Wang H., Zhang S., Li X., Gu B. (2024). The embodied effect in the comprehension of Chinese action-verb metaphors. Journal of Psycholinguistic Research.

[B88-behavsci-16-00914] Winter B., Bergen B. (2012). Language comprehenders represent object distance both visually and auditorily. Language and Cognition.

[B89-behavsci-16-00914] Yao Z., Zhu X. R., Wang Z. H. (2016). The embodied theory of semantic representation: The role of emotion in conceptual representation. Psychological Science.

[B90-behavsci-16-00914] Yu S. Z., Lu S. (2021). A study on the situational systematicity in the embodied semantic processing of L2 abstract verbs. Foreign Language Teaching and Research.

[B91-behavsci-16-00914] Zappa A., Bolger D., Pergandi J. M., Fargier R., Mestre D., Frenck-Mestre C. (2024). The neural correlates of embodied L2 learning: Does embodied L2 verb learning affect representation and retention?. Neurobiology of Language.

[B92-behavsci-16-00914] Zhang S. R. (2021). The embodied effect of metaphor comprehension of Chinese action verbs. Master’s thesis.

[B93-behavsci-16-00914] Zhang X., Yang J., Wang R., Li P. (2020). A neuroimaging study of semantic representation in first and second languages. Language, Cognition and Neuroscience.

[B94-behavsci-16-00914] Zhang Y., Chen S., Peng Y., Yang X., Yang J. (2024). The role of the motor system in L1 and L2 action verb processing for Chinese learners of English: Evidence from mu rhythm desynchronization. Behavioral Sciences.

[B95-behavsci-16-00914] Zhao M. (2022). A study of the effects of word frequency and motor manipulation on the recognition memory of verb-object phrases. Master’s thesis.

[B96-behavsci-16-00914] Zhong M. Y. (2022). The influence of body movements on verb processing. Master’s thesis.

[B97-behavsci-16-00914] Zhu X. T. (2017). An embodied study of motion verbs among Chinese English learners. Master’s thesis.

[B98-behavsci-16-00914] Zwaan R. A. (2021). Two challenges to “embodied cognition” research and how to overcome them. Journal of Cognition.

